# M. Magendie's Experiments on Endosmosis of the Egg

**Published:** 1840-01

**Authors:** 


### M. MAGENDIE'S EXPERIMENT ON ENDOSMOSIS OF THE EGG.

In the review of M. Magendie's Lectures on the Physical Phenomena of Life, in our last Number (p. 318), we referred to an experiment made by that gentleman, as proving what he terms *imbibition by a double current.* This experiment, which appears to us to be novel and ingenious, consisted in placing an egg, with part of its shell removed, in alcohol, so that there should be only the layer of membrane lining the egg-shell between the albumen within and the alcohol without. Supposing at the time, that M. Magendie had used due caution in the performance of this experiment, we gave the result as he states he found it; namely, that while, by *endosmosis*, the alcohol passed through the membrane and coagulated the albumen—by *exosmosis* the albumen itself transuded through the membrane, and mixed with the alcohol in the vessel, rendering it turbid, and forming albuminous flakes. We have lately repeated this experiment more than once; and here, as in other cases, we have not found the result to coincide with the statement of M. Magendie. Having performed it precisely in the manner mentioned by him, we found that while alcohol readily passed in, and coagulated the albumen, in a degree proportioned to the length of contact, not a particle of albumen passed out through the membrane. The alcohol was as clear, after the lapse of a fortnight, as when first placed in the glass. It was allowed to remain until it had sunk, partly by imbibition and partly by evaporation, below the level of the egg; but still no turbidness appeared, nor were any albuminous flakes visible in it. On breaking the egg, a large portion of albumen was found coagulated at the lower part, and firmly adherent to the membrane. The layer of coagulum was dense, and extremely tough.

The experiment was now modified, so as to render the effects visible to the eye. The albumen of an egg was placed in a glass tube, the mouth of which was firmly tied over with a layer of fine skin, so that no liquid could transude, except by imbibition. The tube was then placed with the mouth downwards, in alcohol. In the course of two days the albumen became coagulated within the tube, forming a thick investing layer over the inside of the fine skin, and the action then seemed to proceed but slowly. Still, even after a fortnight's immersion, when the coagulating action seemed to have ceased, the alcohol was perfectly clear and transparent,—not a particle of albumen had passed out. The inside of the skin, on examination, was found to be covered with a dense white layer of coagulated albumen, resembling that found in the egg in the previous experiment.

We can explain the difference in M. Magendie's results only in two ways: 1, We may suppose that he inadvertently punctured the membrane of the egg, in removing the shell; 2, the entrance of the alcohol into the egg forces out, through the small hole made at the opposite end, a quantity of albumen, which drains down, and mixes with the alcohol beneath: if, then, in the experiment, he did not rupture the membrane, we can only refer the difference of his results to this fact, which may have escaped his observation. We have thought it proper to expose this fallacy, since M. Magendie bases many of his speculations on the physical powers of life, on experiments like this, conducted without even ordinary care. It is certain, either that he can have performed this experiment only once; or if more than once, he must have perforated the membrane of the egg each time. How is such an experimentalist to be trusted?

---

